# The University of California San Diego Post-Treatment Glioblastoma (UCSD-PTGBM) annotated multimodal MRI Dataset

**DOI:** 10.1038/s41597-025-06499-z

**Published:** 2026-01-06

**Authors:** Louis Gagnon, Diviya Gupta, Uyen Nguyen, Maria Correia de Verdier, Rachit Saluja, George Mastorakos, Nathan White, Vanessa Goodwill, Carrie R. McDonald, Thomas Beaumont, Christopher Conlin, Tyler M. Seibert, Jona Hattangadi-Gluth, Santosh Kesari, Jessica D. Schulte, David Piccioni, Kathleen M. Schmainda, Nikdokht Farid, Anders M. Dale, Jeffrey D. Rudie

**Affiliations:** 1 Department of Radiology, University of California San Diego, San Diego, USA; 2 Department of Radiology and Nuclear Medicine, Laval University, Québec, Canada; 3 Department of Radiology, University of California Los Angeles, Los Angeles, USA; 4 Department of Surgical Sciences, section of Neuroradiology, Uppsala University, Uppsala, Sweden; 5 Department of Electrical and Computer Engineering, Cornell University and Cornell Tech, New York, NY USA; 6 Department of Radiology, Weill Cornell Medicine, New York, USA; 7 Cortechs.ai, San Diego, USA; 8 Department of Pathology, University of California San Diego, San Diego, USA; 9 Department of Radiation Medicine and Applied Sciences, University of California San Diego, San Diego, USA; 10 Department of Neurosurgical Surgery, University of California San Diego, San Diego, USA; 11 Department of Bioengineering, University of California San Diego, San Diego, USA; 12 Department of Translational Neurosciences, Pacific Neuroscience Institute and Saint John’s Cancer Institute at Providence Saint Johns’ Health Center, Santa Monica, CA USA; 13 Department of Neuroscience, University of California San Diego, San Diego, USA; 14 Department of Biophysics, Medical College of Wisconsin, Milwaukee, Wisconsin USA

**Keywords:** CNS cancer, Cancer imaging

## Abstract

We present the University of California San Diego post-treatment glioblastoma (UCSD-PTGBM) annotated multimodal MRI dataset. The UCSD-PTGBM dataset includes 243 timepoints on 178 subjects with histopathologically-proven glioblastoma who were imaged with an advanced brain tumor protocol on 3 Tesla MRI scanners. Sequences include standard 3D imaging, as well as multishell diffusion (Restricted Spectrum Imaging, RSI) and perfusion imaging techniques (Arterial Spin Labelling, ASL and Dynamic Susceptibility Contrast, DSC), and neuroradiologist approved voxelwise tumor segmentations for both traditional segmentation masks and cellular tumor segmentations. The dataset also includes isocitrate dehydrogenase (IDH) mutation status and O6-methylguianine-DNA methyl-transferase (MGMT) promotor methylation status, as well as overall survival and progression free survival information for a subset of cases. We hope that researchers around the world will use these data to continue to improve analysis of post-operative MRI on glioblastoma patients, translate their findings into clinical practice and improve the management and outcome for these patients.

## Background & Summary

Glioblastoma (GBM) is the most common primary malignant brain tumor in the adult population^1^ and has a very poor prognosis with a median overall survival (OS) of 15–21 months and a 5-year OS of 10%^2^. Treatment for glioblastoma involves a multi-modal approach tailored to the tumor’s characteristics and the patient’s functional status and includes surgery, radiation therapy, and systemic therapies (i.e. chemotherapy, immunotherapy, and targeted therapy). Multimodal MRI is used for evaluating tumor burden, surgical planning, and monitoring disease response to treatment both before and after surgery. Post-treatment imaging of glioblastoma is a fundamental part of patient management that significantly influences clinical decision making and outcomes. The combination of treatment-related changes, including resection cavities, blood products, post-radiation inflammation, and gliosis, combined with the baseline ill-defined tumor borders seen in glioblastomas makes the radiological assessment of post-surgical MRI challenging^3^. Differentiating recurrent disease from post-treatment changes is crucial, since a delay in the diagnosis of tumor recurrence and progression may prevent prompt treatment, while overcalling recurrent tumor may result in unnecessary interventions. Nevertheless, this task remains a challenge even for experienced neuroradiologists.

Over the last 15 years, Machine Learning has opened the door for automated tumor segmentation tools^4^. In parallel, radiomics^5^ has been applied to outcome-related tasks such as overall survival prediction^6,7^, radiogenomics^8^, and differentiating pseudoprogression^9^ from progression based on MRI^10,11^. These models use annotated data that need to be corrected and approved by a radiologist. Most prior research on brain tumor segmentation has been performed in the pre-treatment setting, with a recent survey finding that 98.3% of published glioma segmentation studies had been performed on pre-operative imaging^12^. While several recent studies involving glioma segmentation have been performed in the post-treatment setting^13–21^, relatively little post-treatment glioma data with voxelwise annotations exists publicly^21–24^.

Here we present the University of California San Diego post-treatment glioblastoma (UCSD-PTGBM) annotated multimodal MRI dataset. We aim to provide researchers around the world with a multimodal MRI dataset collected on post-operative glioblastoma patients, and annotated by neuroradiologist. Moreover, our dataset includes advanced imaging such as multishell high b-value diffusion MRI (Restricted Spectrum Imaging, RSI), Arterial Spin Labelling (ASL) perfusion, and Dynamic Susceptibility Contrast (DSC) perfusion sequences. The dataset also includes clinical information such as isocitrate dehydrogenase (IDH) mutation status and O6-methylguianine-DNA methyl-transferase (MGMT) promotor methylation status, as well as overall survival and progression free survival information for a subset of cases. We hope that this dataset will help researchers to improve analysis of post-operative MRI on glioblastoma patients, which could lead to improvement in the management and outcome for these patients.

## Methods

### Patient population and demographic data

This retrospective study was approved by the institutional review board of the University of California San Diego (IRB #809620) and the requirement for informed consent was waived since a significant fraction of the patients had passed away. Data were de-identified and collected according to the Health Insurance Portability and Accountability Act (HIPAA) standard. Only (LG, first author) had control of inclusion of any data and information that might present a conflict of interest. The dataset presented here is the same dataset used in our previous work on cellular tumor segmentation^7^. A subset of this dataset (only the T1 pre-contrast, T1 post-contrast, T2 and fluid attenuated inversion recovery (FLAIR) sequences) was also used in the BraTS 2024 dataset^23^. Patients diagnosed with glioblastoma from January 2010 to June 2022 at UCSD were identified from chart review of the institutional medical record. A drawback of our dataset is the unique institutional origin (UCSD) of all cases, which limits the diversity of imaging protocol and populations. Inclusion criteria included post-treatment MRI with the multishell diffusion sequence RSI. The pathology database was searched to enrich the sample for cases with pathology proven progression and treatment-related changes. 244 unique patients (315 timepoints) were initially identified, but 54 timepoints were rejected due to missing MRI sequences and 18 timepoints were rejected due to inability to distinguish treatment-related changes from residual/recurrent tumor with confidence. The final dataset consisted of 178 patients (243 timepoints) with mean age 56 + /- 13 years [std], 121 men. Data has subject-level identifiers to avoid data leakage. 192 timepoints had residual or recurrent tumor (45 pathology proven), while the remaining 51 timepoints had only post treatment changes (including 4 pathology-proven pseudoprogression (PsP), 9 clinically confirmed PsP, 10 clinically confirmed radiation necrosis, and 28 non-specific post-treatment changes). Overall survival (OS) and progression free survival (PFS) information is available for a subset of 94 patients (58 + /- 13 years, 67 men) with an MRI scan with RSI within 90 days following surgery. IDH mutation status is available for 168 timepoints. MGMT methylation status is available for 149 timepoints. An overview of patient demographics is presented in Table 1.

**Table 1:** Patient Demographics.

	Total	Survival Analysis subset
**Demographics**		
Timepoints	243	94
Patients	178	94
Age (years)	56 ± 13	58 ± 13
Male	116 (65%)	67 (71%)
Female	62 (35%)	27 (29%)
GTR+	87 (49%)	38 (40%)
MGMT+	76/149 (51%)	17/61 (28%)
MGMT−	73/149 (49%)	44/61 (72%)

Note. Data presented as mean ± standard deviation or number (percentage). GTR = gross total resection, MGMT = O^6^-methylguanine-DNA methyl-transferase promoter methylation.

### Imaging data

**Fig. 1:**
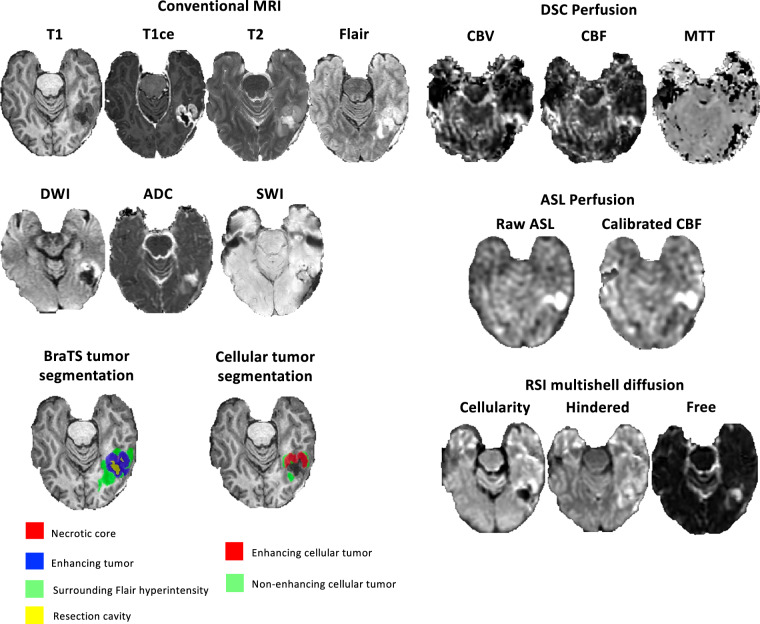
Dataset available MRI sequences and annotations. T1 = T1 pre-contrast, T1ce = T1 contrast-enhanced, FLAIR = Fluid attenuated inversion recovery, DWI = Diffusion-Weighted Imaging, ADC = Apparent Diffusion Coefficient, SWI = Susceptibility-Weighted Imaging, DSC = Dynamic Susceptibility Contrast, CBV = Cerebral Blood Volume, CBF = Cerebral Blood Flow, MTT = Mean Transit Time, ASL = Arterial Spin Labelling, RSI = Restricted Spectrum Imaging.

An example from the dataset is presented in Fig. 1. Imaging protocol included pre- and postgadolinium 3D volumetric T1-weighted inversion recovery spoiled gradient-recalled sequences (TE/TR = 2.8/6.5 ms, TI = 450 ms, flip angle = 8 °, FOV = 24 cm, matrix = 0.93 × 0.93 × 1.2 mm), a 3D T2- weighted FLAIR sequence (TE/TR = 126/6000 ms, TI = 1863 ms, FOV = 24 cm, matrix = 0.93 × 0.93 × 1.2 mm), a T2-weighted sequence (TE/TR = 81.8/5276 ms, 0.47 × 0.47 mm, slice thickness = 3 mm), a susceptibility-weighted sequence (TE/TR = 28/47 ms, 0.47 × 0.47 mm, slice thickness = 3 mm) and a diffusion-weighted sequence (TE/TR = 82/9500 ms, 0.93 × 0.93 mm, slice thickness = 3 mm). For RSI, a single-shot pulsed-field gradient spin-echo echo-planar imaging sequence was used (TE/TR 1⁄4 96 ms/17 s; FOV 1⁄4 24 cm, matrix = 128 × 128 × 48; slice thickness = 2.5 mm) with 4 b-values (b = 0, 500, 1500, and 4000 s/mm2), and 6, 6, and 15 unique diffusion directions for each nonzero b-value, respectively (28 total volumes, ~8 min scan time). DSC perfusion MRI was performed using a gradient-echo echo-planar-imaging (EPI) sequence (TE/TR = 35/1600 ms; FA = 90°; slice thickness 5 mm with intersection gap of 1 mm; 22 axial slices; FOV = 25 cm; matrix = 96 × 96) 0.1 mmol/kg of gadobenate dimeglumine (Bracco Diagnostics) or gadobutrol (Bayer AG), was injected intravenously with an MR-compatible power injector at a rate of 2–3 mL/s through an antecubital angiocatheter, followed immediately by a 20-mL continuous saline flush. The multi-section image set was acquired every 1–2 s during the first pass of the contrast agent until 60 time points were obtained. Aterial Spin Lageling (ASL) with a pseudo-continuous scheme (pCASL) was acquired using the standard GE protocol with in-line analysis. All patients were scanned on 3 T clinical scanners (GE Signa Excite HDx and GE Discovery MR750 with 8-channel head coil) at two different institutions affiliated with UCSD.

### Image pre-processing

The RSI images were processed using a standard processing pipeline described in White *et al*.^25^ to derive cellularity maps. The diffusion signal in each voxel was fit by using a linear mixture model of restricted and hindered water compartments with spheric and cylindric geometries. The resulting spherically restricted water fraction formed the basis of our RSI cellularity maps. To maximize sensitivity and specificity to the spherically restricted water fraction, a beam-forming filter was applied to our cellularity maps to reduce residual signal contamination from cylindrically restricted and hindered water compartments. DSC images were processed using the Matlab DSC toolbox^26^ to compute Cerebral Blood Volume (CBV) using a leakage correction algorithm^27^. Images from all modalities were registered and resampled to a 256x256x256 1 mm isotropic resolution MNI brain atlas^28^. A brain mask was obtained from the FLAIR images using an in-house custom nnUNet skull stripping network and was subsequently applied to the other modalities.

All pre-processing steps were manually reviewed by a neuroradiology fellow (author L.G.) to ensure proper co-registration. Any registration errors were manually corrected before inclusion in the dataset. All the conventional and RSI data were manually reviewed by an attending radiologist during the cellular tumor segmentation procedure (J.D.R.). All the DSC and ASL processed data were manually reviewed (L.G.) and re-processed manually in few cases to ensure good quality.

### Tumor segmentation

#### Standard BraTS segmentation

Multicompartment tumor segmentation was done using the BraTS challenge standard annotation^4^. Image first underwent automated segmentation by a Deep Learning software (OnQ^TM^ Neuro, Cortechs.ai, San Diego). Segmentations were then manually corrected by trained radiologists. Segmentation included four major tumor compartments: enhancing tumor, non-enhancing/necrotic tumor core, surrounding non-enhancing FLAIR hyperintensity, and resection cavity. All manually corrected BraTS-type (enhancing tumor, necrotic core, surgical cavity, FLAIR signal surrounding hyperintensity) tumor segmentations were then reviewed by a board-certified neuroradiologist “approver” (authors J.D.R. and L.G.) in line with the established annotator/approval model used in the BraTS challenges^4,23,29^. In cases where the approver identified an inaccurate or incomplete segmentation, the case was returned to a different annotator for further refinement with notes indicating remaining issues. This process was repeated, if necessary, until the approver judged that the segmentation was accurate. As such, the inter-annotator variability could not be assessed and no cross-validation was performed.

#### Cellular tumor segmentation

For each patient, cellular tumor was manually segmented by an attending neuroradiologist with three years of experience post fellowship and extensive segmentation experience (J.D.R.), as well as a neuroradiology fellow (L.G.). Segmentations were performed based on all available clinical, pathological and imaging history including a thorough review of prior and subsequent MRIs. All cases with post-treatment changes (pseudoprogression, radiation necrosis, and post-treatment changes only) were reviewed by a third neuroradiologist with 13 years of experience post fellowship (N.F.). The neuroradiologists manually segmented total cellular tumor (TCT), representing both enhancing and nonenhancing cellular tumor, while the ground truth enhancing cellular tumor (ECT) was extracted by masking the manual segmentation with the conventional enhancing tumor mask generated as described in the previous section. The non-enhancing cellular tumor (NECT) mask was obtained by masking out ECT from TCT.

## Data Records

This dataset is available through The Cancer Imaging Archive (TCIA)^30^ under a CC BY 4.0 license. The material ca be copied and redistributed in any medium or format for any purpose, even commercially. It is to note that 40 patients (60 timepoints) are held out for the 2025 BraTS challenge and will be released in early 2026. MRI images and tumor segmentations are stored in The Neuroimaging Informatics Technology Initiative (NIfTI) format. Clinical information is stored in a comma-separated values (.CSV) file. Data has subject-level identifiers to avoid data leakage. Data includes T1-pre and post contrast, T2, FLAIR, diffusion-weighted imaging (DWI) and apparent diffusion coefficient (ADC), susceptibility-weighted imaging (SWI), Arterial Spin Labeling (ASL) and corresponding calibrated cerebral-blood-flow (CBF) maps, Dynamic susceptibility contrast (DCS) perfusion imaging (CBV, CBF and mean-transit-time), Restricted Spectrum Imaging (cellularity, hindered, free water).

## Technical Validation

### MGMT promoter stratification

**Fig. 2:**
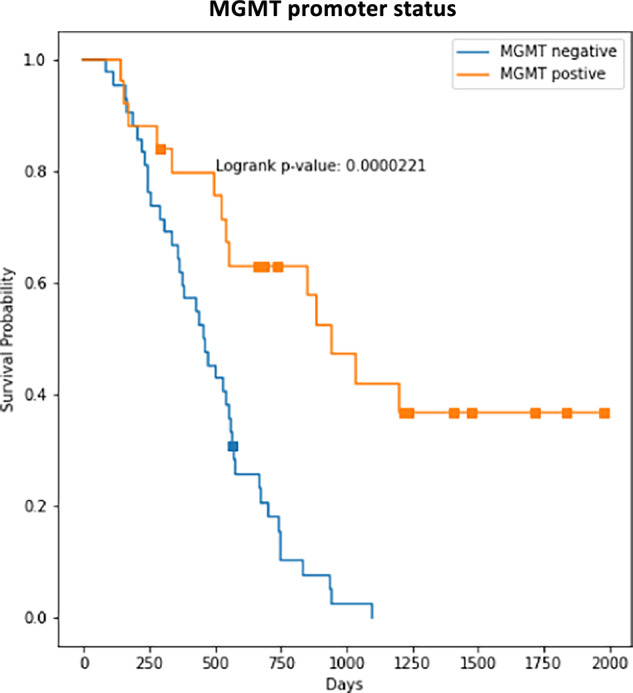
Kaplan-Meier curves for MGMT methylated and unmethylated groups. As expected, patients with MGMT methylation have a significant higher median survival (p = 0.00002).

To demonstrate the internal consistency of our data, we computed the Kaplan-Meier curves for our patient cohort stratified by the MGMT promoter status. Results are shown in Fig. 2. A significant higher (p = 0.00002) median survival time for patients with methylated MGMT status is observed, which is consistent with the literature^31,32^.

## Data Availability

This dataset is available through The Cancer Imaging Archive (TCIA)^30^ under a CC BY 4.0 license (https://www.cancerimagingarchive.net/collection/ucsd-ptgbm/).

## Code availability

The code use to co-register and pre-process the data is available at https://github.com/rachitsaluja/UCSF-ALPTDG-benchmarks15. The code used to process the DSC data is publicly available as part of the Matlab DSC toolbox^26^.
